# Environmental Affordance for Physical Activity, Neurosustainability, and Brain Health: Quantifying the Built Environment’s Ability to Sustain BDNF Release by Reaching Metabolic Equivalents (METs)

**DOI:** 10.3390/brainsci14111133

**Published:** 2024-11-10

**Authors:** Mohamed Hesham Khalil

**Affiliations:** Department of Architecture, University of Cambridge, Cambridge CB2 1PX, UK; mhmhk2@cam.ac.uk

**Keywords:** environmental enrichment, exercise, neuroplasticity, brain health and cognition, architectural design, built environment, spatial layout

## Abstract

**Background/Objectives:** Unlike enriched environments for rodents, human-built environments often hinder neuroplasticity through sedentary lifestyles, to which exercise can merely overcome its adverse effects. This paper introduces "environmental affordance for physical activity" to quantify the potential of spatial layout designs to stimulate activity and sustain neuroplasticity, mainly hippocampal neurogenesis. **Methods:** A novel framework links metabolic equivalents (METs) that can be afforded by the spatial layout of the built environment to its role in increasing the brain-derived neurotrophic factor (BDNF)—a biomarker that promotes and sustains adult hippocampal neurogenesis and synaptic plasticity. Equations are developed to assess the built environment’s affordance for physical activity through BDNF changes measurable after brief exposure to the built environment for 20–35 min. **Results:** The developed equations are evidenced to be feasible to cause BDNF release through low- to moderate-intensity physical activity. This model provides a feasible assessment tool to test the built environment’s effectiveness towards neurosustainability. **Conclusions:** By sustaining neurogenesis, the environmental affordance for physical activity holds promise for improving mental health and preventing cognitive decline.

## 1. Introduction

**Figure 1:**
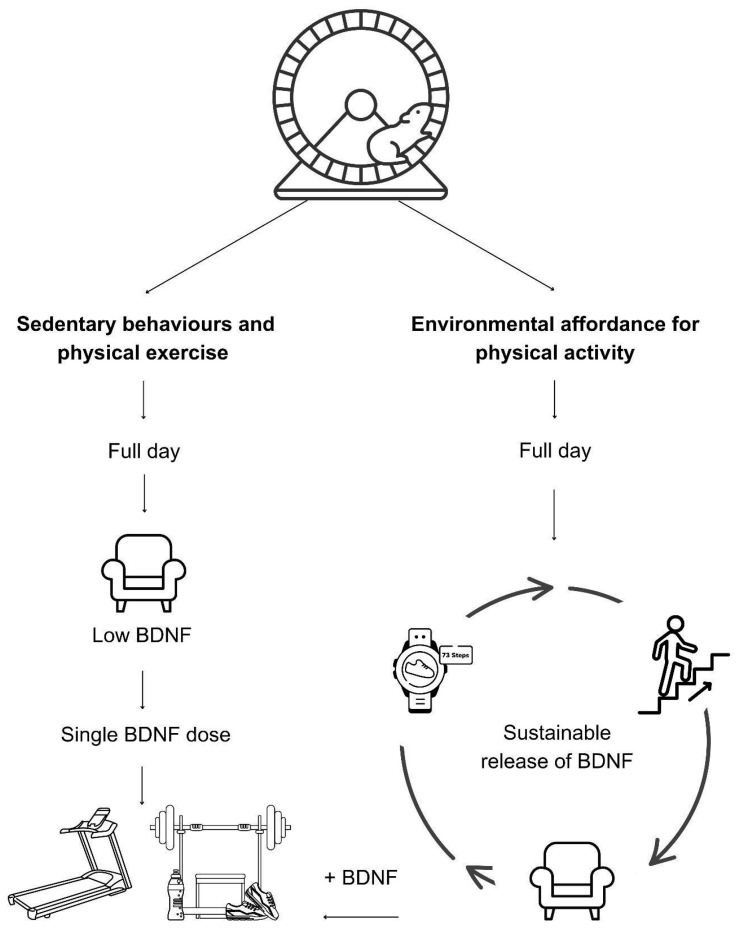
Environmental affordance for physical activity as a sustainable model for neuroplasticity.

Physical activity is considered the critical element for environmental enrichment in rodent studies in the form of running wheels or the additive effect of structural enrichment to the housing cage that promotes physical activity. However, the translation of physical activity into humans’ lifestyles through a sole reliance on physical exercise reveals that the built environment cannot promote physical activity through its design, which can hinder the sustainability of the hippocampal neurogenesis process and cortical plasticity. In that regard, this article introduces environmental affordance for physical activity as a sustainable model for neurosustainability through the built environment. This model was developed upon the premise that physical activity immediately releases the brain-derived neurotrophic factor (BDNF) and other growth factors, which can be measured in blood and are necessary for regulating hippocampal and cortical plasticity processes. This article explores the relationships between architectural design and BDNF through the mediating role of metabolic equivalents (METs) that can quantify the architectural design’s capability for its environmental affordance for physical activity. Figure 1 briefly illustrates the conceptual model before subsequent sections explore and explain its mechanisms at a quantifiable level.

## 2. Physical Activity Mechanisms for Plasticity

Physical exercise has long been considered a critical modulator of adaptive neuroplasticity changes, neurotrophic factors such as BDNF, and improvements in brain function. A recent systematic review of human and animal studies confirmed that physical exercise increases neuroplasticity through the neurotrophic factors (BDNF, GDNF, and NGF) and receptors that provide improvements in neuroplasticity and cognitive function in humans and animals alike [1]. While physical exercise is known to lead to long-term changes in the human brain, specifically in the hippocampus [2,3,4], it is difficult to explain the volume changes and it is more difficult to tell how the built environment can be a successful promoter for the increase in hippocampus volume if changes are measured after several months. Therefore, it is essential to explore the impact of the physical environment’s capability to increase the immediate release of neurotrophic and growth factors that are known to promote and regulate the neurogenesis, synaptic plasticity, and neurosustainability of the multistage plasticity process in the long term in a sustainably consistent manner. Interestingly, biomarkers are more feasible and testable, and some can be representative by measuring only after one trial of less than one hour [5,6]. This section explores the most reliable and evident factors.

### 2.1. Neurotrophins and the Reliable Role of the Brain-Derived Neurotrophic Factor (BDNF)

As mentioned, BDNF is an effective mediator of physical activity’s role in regulating neurogenesis and increasing hippocampal volume, but it may not be the only effective factor.

Until the 1990s, the neurotrophin family was renowned for two factors responsible for the survival and functional maintenance of neurons, the nerve growth factor (NGF) and BDNF, before the identification of neurotrophin-3 (NT-3) in the same family, and later the discovery of neurotrophin 4/5 (NT-4/5) [7,8]. These neurotrophin growth factors provide essential insights into neuroplasticity as they provide important insights into how nerve cells communicate during the development of the nervous system and in neuroplasticity, memory, and learning in the adult nervous system [9].

Regarding the mediating role of the neurotrophins as a result of physical activity, a recent review highlights a positive relationship between physical activity and BDNF levels while highlighting less clear evidence published for the other neurotrophins: NGF, NT-3, and NT-4/5 [10]. The review concluded that 3 months of moderate-intensity exercise, with 2–3 sessions per week lasting no less than 30 min, can be an inexpensive strategy for boosting BDNF release, which this paper sees as comparable to the expected effect of environmental affordance for physical activity, therefore relying on BDNF among the neurotrophins. Another review came to the same conclusion that there is no unanimity in BDNF regulation in humans, unlike in rodents; yet still, BDNF upregulation appears to be in relative agreement, unlike NT-4/5, which displays inconsistent conclusions [11]. The latter review considered articles focusing on training protocols ranging from 4 to 48 weeks in humans, which may explain the lack of unanimity, unlike the other review. More recently, further articles shed light on the effect of the relationship between BDNF and physical activity on humans’ cognitive function and depression [12,13]; both are known to be associated with the level of AHN. A study explores the interactions between BDNF, plasticity, and depression [14]. Therefore, we can expect BDNF to be an effective mediator between physical activity and AHN, as even when hippocampal volume is used as a dependent variable instead of its actual count in humans, the dependencies of increased cognitive function and reduced depression proves its mediating role.

To further explain the mediating role of BDNF in changes in depression and cognition as two proxy measures of AHN, newly formed neurons at different maturation stages may play distinct roles in learning and memory [15]. Disruptions in this neurogenic process have been associated with several cognitive and psychological disorders, including issues with memory encoding, mood disorders, and dementia [16]. Studies found reduced hippocampal volume and decreased neural progenitor cells in the dentate gyrus of humans with major depressive disorder compared to healthy controls [17,18], while antidepressant treatments were found to increase hippocampal volume and promote AHN in animals and humans [19,20]. Additionally, patients with a history of depression were found to have reduced hippocampal activation during a memory task compared to healthy controls [21]. On the other hand, regarding cognitive function, higher levels of AHN are associated with better performance on a pattern separation task [22]. Additionally, post-mortem studies identified that individuals with Alzheimer’s disease (AD) exhibited reduced AHN compared to age-matched controls [23]. Therefore, the mediating role of BDNF between physical activity and AHN in humans can be relied on effectively among all neurotrophins.

The actions of BDNF in neurogenesis and neuronal function, as well as its involvement in the abovementioned pathophysiology and brain diseases, were discussed in [24], who highlight the important role of BDNF in the differentiation, maturation, and synaptic function in the central nervous system. They explained that BDNF stimulates mitogen-activated protein kinase/extracellular signal-regulated kinase (MAPK/ERK), phosphoinositide-3 kinase (PI3K), and phospholipase C (PLC)-gamma pathways mainly through the activation of tropomyosin receptor kinase B (TrkB), which is a high-affinity receptor protein for BDNF that plays a key role in neural development [25]. Another study further elaborates that these signalling pathways contribute significantly to neurogenesis and synaptic plasticity [24].

The effect of physical activity and exercise on BDNF in humans has been extensively reviewed for over a decade. Early in the last decade, a study reported the effect of a single bout of exercise and training on BDNF expression in the brain, in both the working muscles and in the blood, concluding that there are potential benefits to the release of BDNF in the brain and peripheral tissues after exercise [26]. However, they highlighted that the effect on humans required more research. Later, a review revealed through 32 studies that peripheral BDNF concentrations were elevated by acute and chronic aerobic exercise and not through strength training [27]. Afterward, researchers explored in their systematic review, meta-analysis, and meta-regression how different physical exercise parameters modulate BDNF in healthy and unhealthy adult humans, concluding a frequency of 2–3 times per week with an intensity of at least 65% of VO2max (the maximum amount of oxygen that an individual can utilise during intense or maximal exercise) and a moderate-intensity continuous training type for at least 40 min, and noting that BDNF is further improved in lengthier interventions as well as in more intense exercise [28]. Later, researchers explored the effect of physical exercise on neuroplasticity and brain function through a systematic review of both human and animal studies, finding that physical exercise increases neuroplasticity through growth factors, including not only BDNF but also NGF and the glial cell line-derived factor (GDNF), which is shortly discussed subsequently [1]. The authors concluded that physical exercise was effective in increasing the production of neurotrophic factors, cell growth, and proliferation, in addition to improving brain function. Similarly, researchers have explored the physical activity–BDNF-cognition triumvirate, revealing that the literature is heterogeneous [29]. Nevertheless, the specific physical activity type and dose for optimal BDNF release is still unclear. In that regard, researchers conducted a systematic review and Bayesian network meta-analysis on the effects of different physical activities on BDNF, revealing that resistance training > high-intensity interval training > combined training > aerobic training and resistance training > aerobic training alone > control group, suggesting that resistance training at moderate intensity can promote brain health [30]. The review confirms the additive effects of greater intensities on elevated BDNF.

To date, and despite the evidence of the positive effect of physical exercise on BDNF and plasticity in healthy adults, some limitations and gaps still exist. For instance, there is a lack of evidence clarifying the effects of physical activity on neurotrophic factor levels in patients with Parkinson’s disease [31]. Similarly, in middle and late life, researchers found in their systematic review and meta-analysis that there is a significant effect of resistance exercise on insulin-like growth factor 1 (IGF-1), to be discussed subsequently, but not on BDNF, while the effect on the vascular endothelial growth factor (VEGF), to be discussed subsequently, could not be determined due to a lack of sufficient studies [32]. However, another study found an improvement in BDNF among older women, irrespective of exercise type [33]. We can predict that variances among elders are due to the association between age, activity intensity, and type of exercise through the lens of the presented reviews. Most recently, researchers have revealed in their systematic review of 17 articles that aerobic exercise at various intensities, specifically at high intensity, can influence cortical excitability and result in cognitive improvement [34]. The authors highlight the role of exercise on neuroplasticity through the reviewed direct cortical and structural changes. The latter review solidifies the explored role of BDNF and other brain growth factors in mediating the role of physical exercise and neuroplasticity. Regarding hippocampal plasticity, researchers found no effect of aerobic exercise training on hippocampal volume among cognitively unimpaired healthy elders [35]. This is contrary to earlier studies showing that aerobic exercise training increased hippocampal volume compared to control groups [36,37], suggesting that intense physical activity can contribute to enhancing the hippocampal CA2/CA3 volume in young adults [38]. This is in line with the earlier study showing that improving cardiorespiratory fitness after exercise training is associated with an increased volume in the left dentate gyrus/CA3 subregion in young adults [37], which might be associated with neurogenesis as there is an increase in the dentate gyrus volume. This paper concludes that age-related variability needs to be considered when designing and interpreting experimental studies. This can be supported by a systematic review and meta-analysis suggesting that aerobic exercise interventions can help prevent age-related hippocampal deterioration and maintain neuronal health [39]. Another systematic review and meta-analysis revealed that moderate-intensity continuous training and resistance training can augment hippocampal volume to prevent and treat adults with neurodegenerative diseases such as Alzheimer’s disease [40].

This paper considers BDNF the most reliable and effective growth factor mediating the relationship between physical activity and AHN. It can be effectively measured after a single bout of an intervention, yet the long-term effect will always be subject to consistency, intensity thresholds, and duration. Still, this does not impose the use of BDNF to test the effectiveness of environmental affordance for physical activity to increase AHN, hippocampal volume, and BDNF release; reduce depression; and improve cognition in adult humans.

### 2.2. Supportive Growth Factors: VEGF and IGF-1

As evident through the presented state-of-the-art literature, not only is the BDNF from the neurotrophins family an effective mediator between physical activity and neuroplasticity but also other brain growth factors. Little is known regarding the effect of GDNF on neuroplasticity in humans after physical activity. At the same time, the latest meta-analysis and meta-regression shows through the analysis of 11 studies that while BDNF, IGF-1, and VEGF are essential for brain plasticity and cognitive function, physical activity has a significant effect on BDNF and IGF-1 only [41]. Therefore, in addition to the role of BDNF, IGF-1 is also explored. IGF-1 manages the effects of growth hormones (GHs) in the body [42,43], and IGF-1 is part of the insulin-like growth factor family that also includes IGF-2 and others. Both factors and their receptors are widely expressed in nervous tissues, playing a role in developing and maintaining the nervous system, such as in some neurodegenerative diseases and neuroprotective actions [44]. IGF-1 has been shown to have potent effects on central nervous system development, plasticity, and post-injury therapy [45]. One study discussed the role of IGF-1 in hippocampal plasticity after traumatic brain injury [46], while another explored the role of serum IGF-1 in adult human brain morphology [47], proving through magnetic resonance imaging (MRI) that IGF-1 and the growth hormone (GH) contribute to brain growth and that it can be a treatment option for neurodegenerative disorders and brain injury in humans. Interestingly, a study reported that a reduction in insulin-like growth factor family member expression predicts neurogenesis marker expression in schizophrenia and bipolar disorder [48]. A study explains that IGF-1 modulates neuronal excitability and synaptic plasticity at multiple sites, while at the system level, IGF-1 intervenes in energy allocation, proteostasis, mood, cognition, and circadian cycles [49]. With the role played by IGF-1 in neuroplasticity evidenced, it is essential to further investigate its interrelationship with physical activity to achieve the aim of this paper. Researchers conducted a systematic review and meta-analysis on resistance training and IGF-1, revealing that it increases IGF-1 for those who received training for 16 weeks or less among participants older than 60 years old and among women [50]. In their systematic review and meta-analysis, researchers show that IGF-1 serum concentrations are altered by exercise type, yet in conditions that are not well defined, and that there is no determinant in the serum IGF-1 changes for the exercise load characteristics [51]. Regarding the duration, an increase in serum IGF-1, ranging from ~10 to 30%, has been measured after 5–10 min of high-intensity bouts of exercise in healthy individuals [52].

This section concludes that BDNF is the most reliable growth factor among the neurotrophic family and among all other growth factors when it is related to neuroplasticity as a matter of investigation. Exploring the effects of physical activity on BDNF is essential, and exploring it in addition to other growth factors such as IGF-1, VEGF, and others can provide further insights into integrative processes. Testing environmental affordance for physical activity quantified through metabolic equivalent values, which are explored subsequently, is suggested to be achieved through a single bout exposure, which needs careful consideration for the minimum exposure duration and intensity of physical activity through the environment before expecting an increase in BDNF levels or additional growth factors. Various blood test kits can be used to measure BDNF increase/decrease in humans [53]. This paper suggests that coupling the blood test with MRI can provide deeper-level insights [54]. However, it may require longer than a single bout of physical activity if structural changes in the brain need to be observed.

Furthermore, not only can BDNF be associated with improved plasticity at the neurological level, but the role played by the increase in BDNF is promising in multiple mental health and wellbeing issues such as depression [55,56], which is also a proxy measure for AHN, as introduced earlier in this paper, or to hippocampal activity. For instance, a pilot study found that an eight-week exercise intervention improved mood in individuals with major depressive disorder (MDD) but did not enhance memory performance, while both MDD and healthy participants exhibited decreased hippocampal activity, suggesting exercise may increase neural efficiency in low-fit individuals [57]. The authors concluded that exercise promotes neuroplasticity in both healthy and depressed brains, which further affirms the complex interrelationship explored in this paper to urge the design of environments with affordance for physical activity. Additionally, an article explored how environmental factors, particularly exercise and diet, influence neural plasticity through mechanisms involving BDNF signalling, and proposes leveraging these insights to create a “lifestyle pill” for enhancing cognitive function [58]. Thus, environmental affordance for physical activity can be a promising and effective mediator for BDNF, neuroplasticity, and neurosustainability.

## 3. From Physical Activity to Neurogenesis and Plasticity: BDNF Molecular Mechanisms

The key role played by BDNF, from responding to METs achieved by environmental affordance for physical activity to its role in regulating and sustaining neurogenesis and neuroplasticity, is complex and layered. This section explains the molecular mechanisms of BDNF and how it achieves its action on adult hippocampal neurogenesis and neuroplasticity.

It is important to note that BDNF is encoded by the BDNF gene, known for its intricate structure and regulatory mechanisms [59,60]. BDNF is considered a crucial protein in the nervous system, integral to neuronal survival, growth, and synaptic plasticity, but the gene can be read in different ways to produce slightly different versions of the BDNF protein. In other words, the BDNF gene’s ability to produce multiple protein variants enables it to support a wide range of functions in the brain. Depending on what the brain needs at any given time—whether during development, in response to learning new things, or in healing after injury—the gene can produce the specific BDNF most beneficial for those conditions.

Regarding the mechanisms through which BDNF takes place while acting on plasticity in response to stimuli, such as physical activity in this case, BDNF is first synthesised as proBDNF, a precursor that can be cleaved into mature BDNF (mBDNF) [61,62]. mBDNF is essential for promoting neuronal survival and enhancing synaptic connections, directly impacting learning and memory. The conversion of proBDNF to mature BDNF can occur in a relatively short time frame. Intracellular processing by furin happens quickly as part of the secretion pathway.

BDNF exerts its effects primarily by binding to the TrkB receptor, leading to receptor dimerization and autophosphorylation [63]. This binding activates multiple intracellular signalling pathways that promote neuronal differentiation, survival, and synaptic plasticity [64]:

a. MAPK/ERK Pathway: This pathway is involved in cell proliferation and differentiation, as well as migration [65].
b. PI3K/Akt Pathway: Critical for promoting cell proliferation, survival, and metabolism [66].
c. PLCγ Pathway: Involved in synaptic plasticity and neurotransmissions [67].

**Figure 2:**
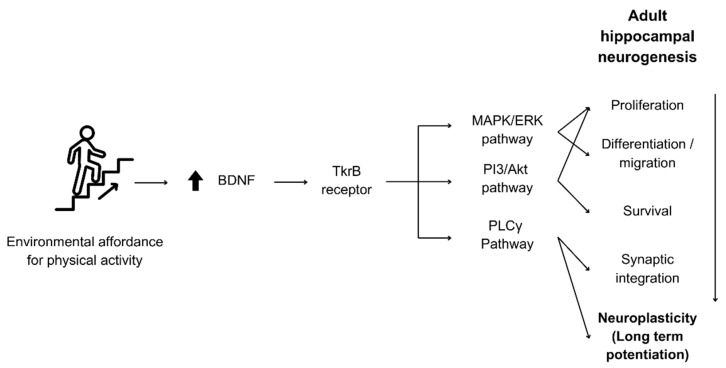
Molecular mechanisms through which BDNF regulates neurogenesis and plasticity by physical activity. Environmental affordance for physical activity increases BDNF levels, which in turn activates the necessary pathways responsible for adult hippocampal neurogenesis and neuroplasticity processes.

These pathways are critical to the perpetual adult hippocampal neurogenesis process from proliferation to differentiation and survival until the synaptic integration of new neurons. In addition to its regulation of neurogenesis, BDNF continues to have effective action on synaptic plasticity after or during synaptic integration, influencing long-term potentiation (LTP), a long-lasting enhancement in signal transmission between two neurons, often considered a cellular correlate of learning and memory, through the activation of MEK-ERK [68]. A summary of how BDNF mediates physical activity to sustain neurogenesis and regulate LTP through the molecular mechanisms discussed is illustrated in Figure 2.

## 4. Methodological Feasibility

**Figure 3:**
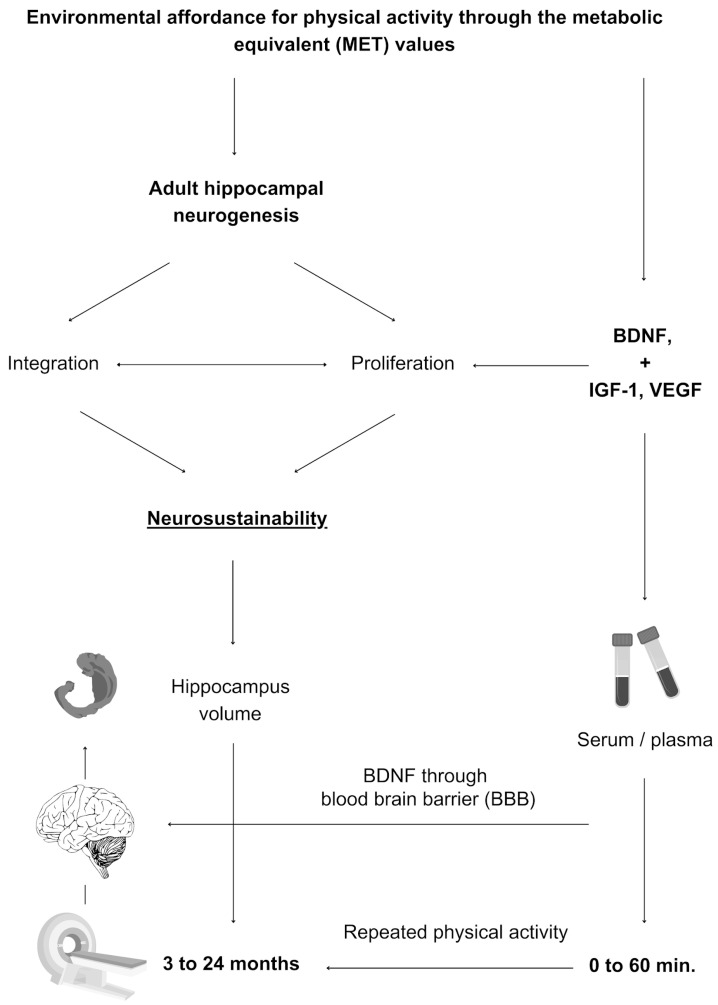
Mechanisms and methods of testing environmental affordance for physical activity. Environmental affordance for physical activity increases growth factors and BDNF (saturated through the blood-brain-barrier) that regulate adult hippocampal neurogenesis and synaptic integration, which leads to a long-term increase in hippocampal volume.

The premise on which the concept of environmental affordance for physical activity is explored subsequently is the feasibility of testing BDNF and other growth factors immediately or in conjunction with long-term hippocampal changes. Figure 3 explains the methodological concept of environmental affordance for physical activity, which is explained in the subsequent section before an assessment tool is developed afterwards.

Physical activity is measurable after a single bout of exposure, while other variables have delayed effects, making it feasible to test environmental affordance for physical activity. A study showed a possible change in BDNF after 20 min of low to moderate gardening physical activity [6]. Similarly, another study exposed healthy older adults to 35 min sessions of physical exercise, cognitive training, and mindfulness practise and then compared the results regarding changes in BDNF levels between the three activities, showing that a single bout of physical exercise had a significantly larger impact on serum BDNF levels than cognitive training or mindfulness in the same person [5]. BDNF level changes from baseline at 0, 20, and 60 min after each intervention ranged between 2 and 3.5 ng/mL for physical exercise, unlike the other two interventions.

BDNF is an immediate and sustainable factor. The immediate release of BDNF is argued to be due to its release in the peripheral nervous system (PNS), but it also releases in the central nervous system (CNS), and BDNF is permeable through the blood–brain barrier [69,70]. Post-exercise BDNF release is also evident in the long term [71], yet it may depend on the physical activity type, and dose of activity [29], intensity [28], and age may be other factors.

Whether to use serum or plasma BDNF is critical to consider in future research. The amount of increased BDNF in the blood can be tested in plasma or serum. Plasma BDNF increased after cognitive exercise and physical exercise (up to 222%) and rest (∼67%), but serum BDNF was elevated only after physical exercise (up to 18%) before returning to baseline after rest. Acute but not prolonged physical exercise increased both plasma and serum BDNF. Cognitive exercise induced acute changes in plasma BDNF only [72].

## 5. Environmental Affordance for Physical Activity: BDNF, METs, and Agents

To increase BDNF release and other growth factors in the human brain, the environment has to afford the induction of metabolic equivalent values (METs), defined as a simple and practical means of quantifying the energy cost of activities [73] in a given point in time through its spatial layout. This paper supports the interrelationship between meeting the MET values and the release of BDNF, where a 20 min low- to moderate-intensity gardening activity intervention activity (mean of 3.5 METs) was sufficient to significantly increase the levels of BDNF as well as the platelet-derived growth factor (PDGF) [6], which is one among multiple growth factors that regulate diverse functions in the central nervous system such as neurogenesis, cell survival, synaptogenesis, and other neuro-related functions. The author later explained in another study that the gardening intervention revealed an association between increased BDNF levels, cognitive ability, and serotonin metabolism, showing the additive effects of the approach [74].

A comprehensive list of the METs of household chores and recreational activities was provided [73], from which this paper derives the means for environmental affordance for physical activity, which is further explored through the existing literature. While the resource examples for heavy housework include grocery shopping, washing floors, washing windows, cooking, ironing, and so forth [73], this paper does not count them as forms of environmental affordance for physical activity because they are not spatially dependent but lifestyle-related. However, walking and climbing stairs are forms of environmental affordance for physical activity, which can be promoted through the design of the built environment.

### 5.1. Walking

Walking was quantified depending on the intensity [73], so this paper adopts the lowest form of walking as 3 km/h, which can be of a light, moderate, or heavy intensity, with MET values of 3, 4, and 5, respectively.

To distinguish between light, moderate, and heavy intensities, the reference distinguished them as when the activity results in only minimal perspiration and only a slight increase in breathing above normal (light intensity), results in definite perspiration and above-normal breathing (moderate intensity), and results in heavy perspiration and heavy breathing (heavy intensity) [73]. This paper supports the argument through a study showing that low-intensity daily walking activity is associated with hippocampal volume in older adults [75], and interestingly, this is in line with the effect of treadmill-walking exercise on hippocampal volume preservation [76]. However, it is worth noting that a study reported that the change in hippocampal volume after 13 months of using treadmills was not mediated by changes in BDNF levels [77]. However, an earlier study explains that inverse correlations turn into positive correlations with all circulating BDNF levels immediately after exercise [78]. This suggests that neuroplasticity-related changes in the hippocampus and BDNF increases are not synchronous. In other words, it is suggested that measuring BDNF after walking should take place immediately after the walking activity, while measuring hippocampal neuroplasticity does not have an immediate result. This distinction further supports the suggested role of BDNF as one among multiple brain growth factors that promote neurosustainability by facilitating the neuroplastic changes themselves.

The MET value through walking primarily depends on the scale of the environment, whether urban or interior. The former abundant navigation networks, unlike the latter, but knowing that people spend the majority of their time inside built environments still poses a great challenge [79]. The extent to which walking can be of a light, moderate, or high intensity is arguable due to extended walking, which might be more feasible in the urban rather than the interior environment.

Several variables can influence walking at the urban level. For instance, street features are found to shape pedestrians’ leisure walks in cities [80], and green space has an effect on walking as well [81]; people can also be nudged into physical activities through minor changes to the urban landscape [82]. This is a non-exhaustive list of potential variables that we argue in this paper to be a necessary agent for increasing walking as necessary for releasing BDNF in the human brain.

At the interior level, we argue that certain design features can play an important role in influencing walking as well depending on the building type and the layout design. Different types of utilitarian walking, such as walking to other activities, to a front entry, and so forth, are influenced by design features but marginally influenced by the number of storeys of the building [83], which we explore subsequently. A systematic review found that increasing opportunities for walkable spaces and reducing physical barriers can result in higher levels of physical activity among the elderly [84].

Regarding residential buildings, increasing the area through the spatial layout design may not be merely effective for promoting physical activity. For instance, in a study conducted on rodents, merely increasing the housing size is proven not to increase physical activity [85]. This is in line with research on humans, where a study found that apart from house size, families spend the majority of their time at home in the same space [86]. The antidote to promote walking is argued to be through the spatial layout. A recent study also shows that residential buildings with traditional house layout patterns appear to have an advantage over modern or Western styles regarding spending the least time on sedentary activity due to the fact that traditional houses are designed with the separation of living and sleeping areas from kitchens, toilets, and bathrooms through the presence of courtyards and corridors, unlike Western modern houses that are designed with integrated areas [87]. Therefore, architectural design needs to critically consider how to encompass environmental affordance for physical activity through layout design in order to increase step count inside homes, workspaces, and other building uses in order to meet at least the minimum METs for walking, especially since counterbalancing the effect of sedentary behaviours at home is mainly explored through the lens of exercise equipment [88].

Last but not least, a recent study found that employees in workplaces with linear layouts tend to have higher levels of physical activity than those in radial layouts [89].

### 5.2. Climbing Stairs

Using the stairs was quantified as of light, moderate, or heavy intensity, with MET values of 4, 6, and 8, respectively [73]. The distinction between the three forms of intensity is similar to how it is explained in the previous section.

There are no studies in the literature regarding neuroplasticity or BDNF release after the immediate or long-term habit of climbing staircases, but several studies indicate that there is a gap rather than a lack of evidence. For instance, studies indicate that short bouts of stair climbing in a naturalistic setting and brief stair-climbing interventions can induce cognitive benefits and mood states [90,91], which suggests that it is an effective means for environmental affordance for physical activity, and in this paper, we argue that climbing staircases can have a greater effect than walking, given its higher MET values. A study discussed that exercises of ≤1 min bouts throughout the day can improve cardiorespiratory fitness and reduce the negative impact of sedentary behaviours, relying on more evidence on the benefits of climbing stairs that aligns with this argument [92]. Most recently, a randomised controlled crossover trial demonstrated that interval stair climbing can confer immediate psychological benefits [93], providing further evidence that climbing stairs is a promising means to address physical inactivity issues, which this paper takes as additional support for using stairs as a structural form of environmental enrichment to satisfy environmental affordance for physical activity to release BDNF and further promote neuroplasticity.

The MET value by climbing stairs can primarily depend on the heart rate induced, potentially by the frequency of using the staircase in a fixed time and also arguably by the staircase design. As mentioned above, stair climbing can have low, moderate, and high intensities, which result in various METs; we explained that it is dependent on indicators such as breathing, which is well studied through, for instance, heart rate [94,95,96,97]. This method should help with identifying the mean value of the MET, to be used to predict the change in BDNF levels, which will be explained later in this article.

Regarding the influence on the use of vertical circulation, evidence suggests that once the destination was in sight, regardless of whether it had high or bare visibility, participants made an instantaneous decision to switch floors and move up toward the destination [98], which we argue can be a promoter for staircase use to release BDNF by meeting the MET values. However, it is crucial to consider that the interaction between visibility and prior background expectations affects wayfinding efficiency and strategy during vertical navigation tasks [99], which we suggest to be taken into consideration in the experiment design process when people are familiar with the environment. These studies offer valuable insights into initial calls for research and interventions on the influence of building design and site design on physical activity by considering stairs, layout, and building form [100]. A previous study’s authors discussed the need to provide staircases in homes as a requirement for MET-based physical activity [79]. Hence, this paper concludes that the design of the layout both horizontally and vertically may lead to various extents of overcoming sedentary behaviours by increasing environmental affordance for physical activity to release BDNF in the human brain.

### 5.3. Environmental Agents

Table 1 synthesises key findings from the previous section regarding the design and quantification of environmental affordance for physical activity. We explained that the intensity of physical activity can be identified through heart rate measures and can be influenced by several built environment factors at the architectural and urban levels.

**Figure 4:**
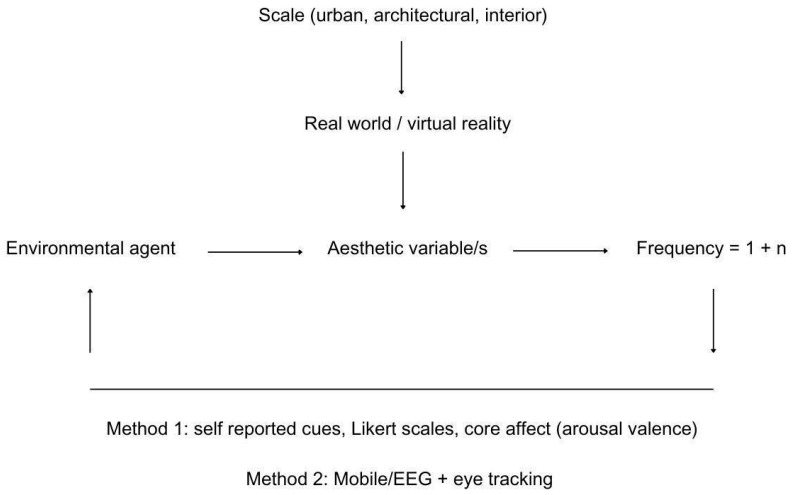
Framework and potential mixed methods for exploring visual environmental agents.

Certain environmental agents can drive the use of the environment, after its affordance for physical activity, not only through structural enrichment but also through visual cues. For example, certain aesthetic variables (such as hominess or fascination) may affect environmental use differently [101,102]. We expand the exploration of environmental agents to include elements in the environment that may drive environmental use and may or may not be associated with the aesthetic variables per se. For example, if natural stimuli influence environmental use rather than artificial ones, they may or may not be associated with an aesthetic variable such as hominess or fascination. In that regard, Figure 4 illustrates the linear relationship expected between environmental agents (natural or artificial) and aesthetic variables, as well as increases in frequency depending on the dynamics between the two former variables. It also explains potential methods either using Likert scales and core effects, as found to be effective in recent studies [102], or through combined Mobile/EEG and eye tracking, which can be more insightful [103,104,105]. Testing this model should be feasible through real-world experimentations or virtual reality [106,107].

While this paper does not intend to limit the breadth of potential environmental agents, some examples are given to guide future research at this stage. At the broader level, environmental agents can be natural or artificial, yet both can be aesthetic in essence. Visual cues found in nature can feature variables such as dynamism, movement, rhythm, unpredictability, and restorativeness, while human-made variables can have a greater depth of meaning, such as artwork, with an unlimited breadth of interpretations. Within these two categories, individuals can have various responses to the perceived hominess or fascination, for instance, which may drive the use of a layout with environmental affordance for physical activity. This is an interesting and open-ended area of exploration.

## 6. Assessment Tool

There are various ways to test if the urban or architectural design has the potential to hold environmental affordance for physical activity in order to promote neurosustainability through BDNF release. Some studies are able to isolate the effect of walking from the effect of climbing stairs depending on the scale (urban and architectural) and also depending on the building type. However, at the architectural level, the effect of the layout design on the extent to which a design holds a potential for environmental affordance for physical activity will rely on the combined effect of the intermittent use of walking and climbing stairs. In that regard, Equations (1)–(3) explain the assessment tool based on the expected constant (α), relating METs and time (*t*) to the increase in BDNF based on each case and if combined together.

(1) $$BDNF_{stairs}=\alpha \times MET_{mean\;intensity}\times t$$

(2) $$BDNF_{walking}=\alpha \times MET_{mean\;intensity}\times t\;\;$$

(3) $$BDNF_{combined}=\alpha \times \left(\sum \;\left(MET_{mean\;intensity}\times t\right)\right)\;\;$$

Equation (4) aims to help with understanding the effect size of an urban or architectural design on the increase in BDNF from the baseline compared to a control group. However, it is important to note that the BDNF level post-test varies from the immediate effect with intervals up to one hour [5]. Testing changes in BDNF may be most effective at the immediate level, but it is important to generalise the time at which blood samples are taken from all participants. One study showed that 35 min of physical activity did not yield much difference from cognitive stimulation or mindfulness training [5], which can help with isolating the effect of environmental affordance for physical activity on BDNF change.

(4) $$BDNF_{adjusted}=BDNF_{baseline}+BDNF_{posttest}$$

Regarding expected time of exposure, two studies report that 20 to 35 min of physical activity of low to average intensity is sufficient to cause changes to BDNF levels [5,6], which facilitates taking the mean value and conducting experiments with control and experiment groups without time-consuming exposure.

## 7. Future Research Directions

The developed methodological means, theoretical framework and assessment tool for environmental affordances for physical activity are promising to be of interest to researchers studying the impact of physical activity on public health. This is because engaging with the environment can sustain adult hippocampal neurogenesis, which continues into a person’s eighth and even tenth decade [108,109].

Testing neurogenesis in humans is less feasible than in animals due to methodological ethics, which makes shifting focus on boosting the release of BDNF in humans through environmental enrichment of utmost importance. BDNF is known to mediate physical activity-dependent neurogenesis [110,111]. The contribution of this paper is in line with the increasing interest in the relationship between BDNF and sedentary behaviours. Among multiple studies, BDNF was recently found to be associated with sedentary patterns in patients with type 2 diabetes mellitus [112], and is currently being reviewed as inversely associated with such sedentary behaviours among children and adolescents [113], which makes the contribution of this paper with a model for an integrative framework a critically needed antidote for the modern environment to ensure the neurosustainability of the human brain [114].

This paper provides a stepping stone for future research aiming to counterbalance the adverse effects of modern environments on the human brain, where sedentary behaviours impose, among other factors, a dead end for neuroplasticity [115,116,117,118,119,120,121,122,123].

The theoretical model developed in this paper supports the need for intuitive innovation that may not yet be fully recognised as a formal area of scientific research but is considered as critically needed to address complex global challenges through a more open, interdisciplinary approach to scientific inquiry through the exploration of unconventional forms of knowledge generation [124]. Despite the promising approach of every theoretical innovation, some challenges are worth noting at this point:

1. Age differences correlate with neurogenesis rate variances and potentially variant impacts on BDNF change rates. For instance, several studies reported an age-related decline in adult hippocampal neurogenesis in humans despite the quantified rate per day, and that this decline varies between healthy individuals and patients with Alzheimer’s disease [109,125,126]. Therefore, controlling for age group variances should be considered in future research.
2. BDNF does not solely respond to physical activity but to many biological and lifestyle confounding variables. Besides ageing and neurodegeneration, sleep [127], stress [128,129], and diet [130] are factors that can have confounding effects. In a recent study, participants were required to fast for 9 h before blood sampling prior to starting a 20 min gardening activity [6], which can be an appropriate way to control diet-related factors in addition to adequate sleep.
3. Measuring BDNF in real-world settings may be sensitive to temperature, heat, and seasonal variations. Seasonality variations and heat exposure were reported to affect BDNF levels, as observed in several studies [114]. Therefore, it is essential to control for such external confounding effects.
4. Longitudinal effects of BDNF changes hippocampal volume are analysed using MRI brain scans after an average of 3 to 6 months, while recent trends lean towards the analysis of the post-mortem tissue as an alternative means depending on the population and ethical considerations [131,132].
5. Salivary BDNF methods should be employed cautiously. BDNF results were not reliable in saliva in three different commercial ELISA kits [133].
6. It is feasible to test changes in BDNF release in serum or plasma, with concentrations in the former about 100-fold higher than the latter [54].
7. Ethical approvals for blood samples to test BDNF changes may only limit non-interdisciplinary experimental efforts without collaboration with at least one researcher with a background in biology or medicine. This encourages interdisciplinary collaborations or otherwise relies on proxy measures such as physical activity-dependent heart rate and skin conductance changes through wearable devices [134].
8. This article highlights that while determining the factor (α) is a preliminary step before attempting to test the impact of spatial layouts with walking and climbing affordances, the factor itself for stairs, for instance, may vary depending on the type of staircase (i.e., straight, with intermediate landing, l-shaped, double l-shaped, u-shaped, 90 winder, 180 winder, curved, and spiral stairs), the number of flights, and the riser’s height. Such variability can affect the intensity that METs already acknowledge, but having a constant (α) for each staircase type may be a limitation that may be overcome to save time replicating the same experiment. The same concept applies to layouts with various areas and spatial division approaches. Unless no combined effect of walking and climbing is to be studied, the constant (α) may not pose a limitation.
9. Individual differences, personality traits, and lifestyle variability may influence the interaction with a layout with environmental affordance for physical activity and the interaction with visual environment agents due to the meaning-making variability as part of any aesthetic experience [135]. Overcoming this limitation can be achieved through controlling cultural and social differences.

These challenges are seen to open more paths to additional research efforts to bridge methodological, environmental, and individual differences, which can speed the process of exemplifying environmental affordance for physical activity towards lifelong neurosustainability.

This paper also follows up on recent evidence suggesting that spending extensive time inside built environments, specifically homes, has adverse effects on cognition [136], suggesting that environmental affordance for physical activity can reduce the negative impact of built environments on brain health. This paper adds another dimension to the recent evidence, showing that natural environments perform better than built environments in response to stress [137,138], stressing that physical activity can be another critical variable to consider in the built environment, along with recent suggestions about biophilia integration. Environmental neuroscience, interested in understanding how physical environment ingredients impact mental health [139], can highly benefit from the approach introduced in this article.

## 8. Conclusions

**Figure 5:**
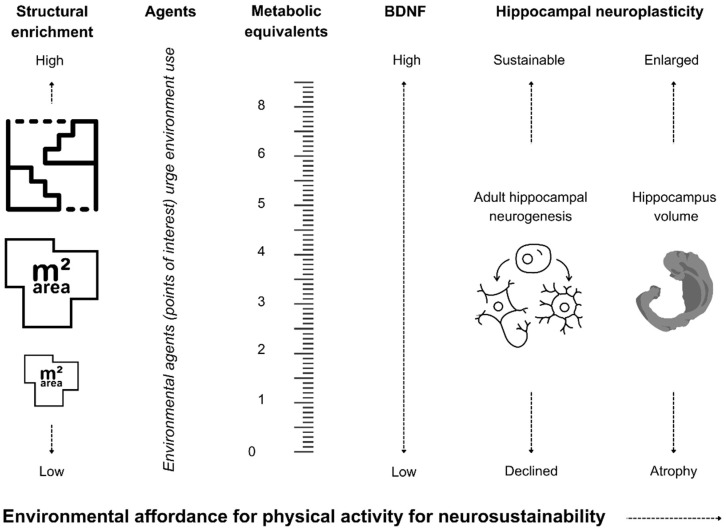
Summary of environmental affordance for physical activity mechanisms for adult hippocampal neurosustainability.

This paper proposes environmental affordance for physical activity, as summarised in Figure 5, as a sustainable model for regulating hippocampal neurogenesis and plasticity processes in the human brain by meeting MET values through the spatial layout’s structural enrichment in buildings by architectural design. Knowing that the process of neurogenesis persists until the tenth decade of life and that measuring neurogenesis in humans is difficult, testing environmental affordance for physical activity and easily measuring BDNF in serum or plasma immediately after a single bout of using the enriched environment is highly promising. The developed equations, framework, and methodological guidelines facilitate the use of this assessment tool to tell if the environment, urban or architectural, is designed with the ability to stimulate BDNF release in the human brain. This should help overcome not only sedentary behaviour-related health issues but also various negative psychiatric and neurological problems in the modern era associated with the hippocampus function, such as pattern separation, depression, anxiety, and impaired cognition.

## Figures and Tables

**Table 1:** Environmental affordance for physical activity through metabolic equivalents (METs).

Structural Enrichment	METs per Intensity			Built Environmental Agents	
	Light	Moderate	High	Architectural	Urban
Walking 3 km/h	3	4	5	e.g., division of space, zoning, long corridors, linear layouts,…, etc.	e.g., street features, green space, routes.
Climbing stairs	4	6	8	e.g., staircase type, floor visibility, novelty.	N/A
Combined METs_mean_	3.5	5	6.5		

## Data Availability

No new data were created or analysed in this study. Data sharing is not applicable to this article.
